# Electrolyte-Dependent Modification of Resistive Switching in Anodic Hafnia

**DOI:** 10.3390/nano11030666

**Published:** 2021-03-08

**Authors:** Ivana Zrinski, Cezarina Cela Mardare, Luiza-Izabela Jinga, Jan Philipp Kollender, Gabriel Socol, Alexey Minenkov, Achim Walter Hassel, Andrei Ionut Mardare

**Affiliations:** 1 Institute of Chemical Technology of Inorganic Materials, Johannes Kepler University Linz, Altenberger Str. 69, 4040 Linz, Austria; ivana.zrinski@jku.at (I.Z.); cezarina.mardare@jku.at (C.C.M.); jan.kollender@empa.ch (J.P.K.); achimwalter.hassel@jku.at (A.W.H.); 2 Danube Private University, Steiner Landstrasse 124, 3500 Krems-Stein, Austria; 3 National Institute for Lasers, Plasma and Radiation Physics, Atomistilor Str. 409, 077125 Bucharest-Magurele, Romania; izabela.jinga@inflpr.ro (L.-I.J.); gabriel.socol@inflpr.ro (G.S.); 4 EMPA, Laboratory for Joining Technologies & Corrosion, Swiss Federal Laboratories for Materials Science and Technology, Überlandstrasse 129, 8600 Dübendorf, Switzerland; 5 Christian Doppler Laboratory for Nanoscale Phase Transformations, Center of Surface and Nanoanalytics, Johannes Kepler University Linz, Altenberger Str. 69, 4040 Linz, Austria; oleksii.minienkov@jku.at

**Keywords:** memristor, anodic oxide, hafnium oxide, valve metals

## Abstract

Anodic HfO_2_ memristors grown in phosphate, borate, or citrate electrolytes and formed on sputtered Hf with Pt top electrodes are characterized at fundamental and device levels. The incorporation of electrolyte species deep into anodic memristors concomitant with HfO_2_ crystalline structure conservation is demonstrated by elemental analysis and atomic scale imaging. Upon electroforming, retention and endurance tests are performed on memristors. The use of borate results in the weakest memristive performance while the citrate demonstrates clear superior memristive properties with multilevel switching capabilities and high read/write cycling in the range of 10^6^. Low temperature heating applied to memristors shows a direct influence on their behavior mainly due to surface release of water. Citrate-based memristors show remarkable properties independent on device operation temperatures up to 100 °C. The switching dynamic of anodic HfO_2_ memristors is discussed by analyzing high resolution transmission electron microscope images. Full and partial conductive filaments are visualized, and apart from their modeling, a concurrency of filaments is additionally observed. This is responsible for the multilevel switching mechanism in HfO_2_ and is related to device failure mechanisms.

## 1. Introduction

Memristive effects in various materials are continuously reported and the interest for memristive devices grows due to their wide range of applications. Redox-based random access memories (ReRAMs), as a new generation of memories more progressive than FLASH technology, and dynamic random access memories (DRAM) are some of the most common applications [1,2,3]. Additionally, memristors are used as building blocks for neuromorphic architectures [4] such as artificial synapses [5] and logic circuits due to the possibility of multilevel switching to implement a fuzzy behavior [6,7,8]. Due to their reported high stability, memristors are also used for various sensing applications [9]. Generally, the main memristive effect research is focused on ReRAMs [10] in metal-insulator-metal systems (MIM) [11], showing non-volatile properties [12,13]. The inherent operational memory of the device is based on conductive filament (CF) [14] formation or deletion during SET or RESET processes enabling switching between high resistance states (HRS) and low resistance states (LRS) when biased at voltages of different polarities [6]. Solid-state redox reactions within such structures become possible due to the electrical field activated migration of vacancies and ions accompanied by electron fluxes. Generally, CF formation is regarded as the active mechanism based on partial reduction of solid electrolyte forming local oxygen deficient regions with their charge compensated by electrons [15]. However, there is a possibility that mobile cations are accompanying this process, including other species that can be trapped inside the memristive layer [3,7,10,16].

The strongest candidates as materials for memristors fabrication are valve metals such as Ti, Ta, Nb, or Hf [17,18,19]. Hafnium dioxide is typically a compact oxide with a high dielectric constant, large bandgap, and high chemical and thermal stability [20]. Anodic formation of Hf oxide directly on Hf parent metal thin films already showed admirable electrical properties for electronic applications [21,22]. Therefore, the use of inexpensive, simple, and fast electrochemical methods for fabrication of the oxide layer necessary in MIM structures such as Pt/HfO_2_/Hf may present some advantages for being used in ReRAMs competing with more expensive fabrication methods such as sputtering [18,23,24]. The aim of this work is to assess the stability, reproducibility, and feasibility of anodic memristors on Hf. The influence of phosphate (PB), borate (BB), and citrate (CB)-buffered electrolytes used for anodization on the final memristive behavior of HfO_2_ is studied. Questions leading to understanding of switching mechanism are raised as linked to fundamental knowledge of electrochemical parameters influence on the final oxide behavior. This can further advance current theoretical models of Hf oxide memristors switching modes as a necessary step in answering the very actual question of physical CFs formation and their reversibility [25,26,27].

## 2. Materials and Methods

### 2.1. Fabrication of Anodic HfO_2_ Memristive Devices

Thin films were sputtered from a high purity Hf target (99.95% Demaco, The Netherlands) on previously thermally oxidized Si wafers at 950 °C during 24 h. An ultra-high vacuum system (Mantis Deposition, UK) with a base pressure in the range of 10^−6^ Pa was used for this purpose. These films were the parent metals to be anodized in a following fabrication step. Deposition of Hf by sputtering was done at room temperature in Ar atmosphere with a pressure of 5 × 10^−1^ Pa. The final thickness of the parent metal Hf film serving as bottom electrode was 350 nm. In order to obtain an oxide layer (HfO_2_), samples with formed Hf films were electrochemically anodized in a three-electrode system at ambient conditions. In the electrochemical cell, the Si wafer with SiO_2_ layer (thickness ≈ 500 nm) and Hf thin film was placed as the working electrode, and a graphite foil of 0.5 mm thickness (99.8% ThermoFisher, Erlangen, Germany) was the counter electrode, whereas the Hg/Hg_2_SO_4_/sat. K_2_SO_4_ electrode (0 V vs. Hg/Hg_2_SO_4_ = 0.640 V vs. SHE) was the reference electrode. The experiments were accomplished using a CompactStat potentiostat (Ivium Technologies, Eindhoven, The Netherlands). The oxides were grown potentiodynamically at a scan rate of 100 mV s^−1^ up to 8 V (vs. SHE) using different electrolytes. Anodic oxide films with thicknesses in the range of 17 nm were obtained in this way. Firstly, the anodization process of Hf films was done in 1 M phosphate buffer with a pH of 7.0. Then, separate samples were anodized in citrate buffer 0.1 M with a pH of 6.0 and 1 M borate buffer with a pH of 9.0. All electrolytes were prepared with ultrapure water (Millipore) and all used chemicals (Na_2_HPO_4_, NaH_2_PO_4_, H_3_BO_3_, NaB_4_O_7_, C_6_H_9_Na_3_O_9_, C₆H₈O₇) were of analytical grade (Merck, Darmstadt, Germany), used without any further purification [28]. Final stages of memristive device fabrication consisted of sputtering 100 nm thick Pt top electrodes 200 µm in diameter. This was done using a high purity target (99.95%, MaTeck, Jülich, Germany). Electrodes patterning was performed in Ar atmosphere (5 × 10^−1^ Pa) at room temperature through a pre-attached 30 µm thick Ni shadow mask foil (Mecachimique, Pierrelaye, France). This was placed in intimate contact with the HfO_2_ surface and allowed patterning of more than 300 top electrodes on a single wafer. For ensuring thin film uniformity for all sputtered layers, all samples were rotated at 5 rpm.

### 2.2. Electrical and Thermo-Electrical Measurements

A unique experimental configuration was used for the purpose of electrical parameters investigations including *I-U* sweeps, endurance tests, retentions tests, and thermo-electrical measurements. A Gantry robot developed in-house with high precision XYZ translation stages is responsible for connecting a W needle (with a tip diameter of 10 µm attached to the Z stage) to top electrodes of memristors under test. Their precise positioning was performed with the aid of two microscope cameras (Bresser, Rhede, Germany) enabling viewing angles of 45° and normal to the Pt electrodes. The whole setup was connected to a Keithely 2450 SourceMeter Unit parameter analyzer. A force sensor was responsible for keeping the contacting force at a constant value of 20 ± 2 mN. In addition, the bottom metallic Hf film at the edge of Si substrates was contacted by a stainless-steel needle. Self-developed LabView^®^ software was specially designed for each group of tests that were all realized in ambient conditions (22 °C, 55% RH), and the voltage was biased against the Hf bottom electrode while the Pt top electrode was grounded. In order to form memristors, the MIM structures were biased in positive direction up to a voltage value reaching approximately half the formation potential used in oxide layer fabrication via anodization (8 V was applied to obtain 17 nm thick oxide layer). In a second step, the devices were switched in a cycle (i.e., from 0 V to 4 V, 4 V to −4 V, and back from −4 V to 0 V). Generally, the current compliance was in the mA range and switching voltages *U*_set_ and *U*_reset_ were between ±0.5 and ±4.0 V. A sweep rate of 230 mV/s was used for memristors formed in PB, CB, and BB in this range (±4.0 V for PB, ±2.0 V for BB and CB). Finally, endurance and retentions tests were completed up to 10^6^ cycles by biasing memristive devices at their switching voltages (*U*_set_ and *U*_reset_) with the fixed current compliance used during *I-U* sweeps. The device resistance was read at 0.02 V within 3.8 ms.

Thermo-electrical measurements were done in the same conditions although under the Si wafer holder on mobile XY stage of the Gantry robot, a round electrical heater 100 mm in diameter encapsulated in an Al_2_O_3_ block was positioned. Once memristors were formed (at room temperature), the effect of the temperature was studied by heating the samples stepwise from 22 °C to 100 °C and recording *I-U* sweeps at each step. Then, endurance testing was performed at selected temperatures where each sample (PB, BB, CB) showed the best switching characteristics (highest HRS/LRS ratios). Finally, memristive switching at the best performing temperature was investigated and retention of HRS and LRS was studied for cycles up to the failure limit. The resistance for both retention and switching test was read at 0.02 V. Impedance spectroscopy was applied for investigating the resistive and capacitive properties of memristors grown in different electrolytes in different states (LRS and HRS). A sinusoidal AC excitation signal with an amplitude of 100 mV was applied for this purpose while the frequency was varied between 100 kHz and 1 Hz. A Compactstat potentiostat (Ivium Technologies, Eindhoven, The Netherlands) operated in 2 electrode configuration was used to perform the impedance measurements of the solid-state devices. All measurements were performed at 0 V bias.

### 2.3. Imaging and Analysis Methods

High resolution (HR) transmission electron microscopy (TEM) was performed on memristors using a JEM-2200FS (JEOL, Tokyo, Japan) equipped with in-column Ω-filter and operated at an acceleration voltage of 200 kV. Cross-sectional samples were prepared via focused ion beam FIB Crossbeam 1540XB (Zeiss, Jena, Germany) operated with Ga ions at 30 kV and 5 kV for cutting and thinning, respectively. For elemental characterization of specimens, energy dispersive X-ray spectroscopy (EDX) mapping was utilized using a detector from Oxford Instruments (UK) and Aztec software. The Gatan DigitalMicrograph*™* software was routinely applied for (HR)TEM images processing. The chemical composition of anodic memristors grown in different electrolytes was evaluated by X-ray photoelectron spectroscopy (XPS). All measurements were performed with ESCALAB Xi+ (Thermo SCIENTIFIC Surface Analysis, East Grinstead, UK) equipped with a multichannel hemispherical electron analyzer (dual X-ray source) working with Al K_α_ radiation. As energy reference, C 1s was used with a binding energy of 284.8 eV. The surface chemical compositions and oxidation states were obtained from the XPS spectra by calculating the integral of each peak after subtraction of the “S-shaped” Shirley-type background using the appropriate experimental sensitivity factors by means of the Avantage software (version 5.978). The spectra were analyzed using NIST X-ray Photoelectron Spectroscopy Database and The Handbook of X-ray Photoelectron Spectroscopy [29]. Depth profile investigations were performed by quantitative analysis after Ar^+^ sputtering of the surface in a 2 × 2 mm^2^ spot. The energy used for sputtering was 2 keV and was applied for three periods of time corresponding to depths of 2, 6, and 12 nm. XPS high resolution valence band spectra were performed in the 0–40 eV energy range, with a pass energy of 20 eV, a dwell time of 50 s, and number of scans equal to 120.

## 3. Results and Discussion

### 3.1. Anodic Memristors Structure and Composition

**Figure 1:**
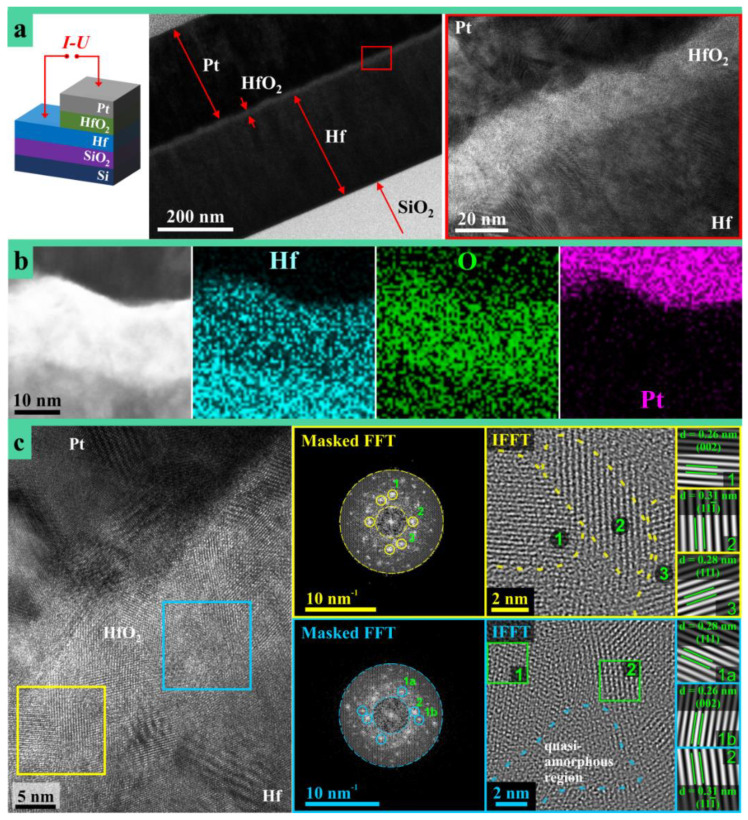
(**a**) Schematic description of the anodic memristor structure (grown in phosphate (PB)) with cross-section imaging for thickness observation and zoomed-in TEM view of the anodic oxide between Pt and Hf metallic electrodes. (**b**) Chemical analysis map (reconstructed using L series of characteristic X-Ray spectrum for Hf and Pt) of a small region FIB-cut through the anodic memristor (grown in PB) showing the species location in the device. (**c**) (HR)TEM imaging of an anodic memristor (grown in PB) along with masked FFTs of regions highlighted with blue and yellow frames. Section (**c**) also contains filtered IFFT and IFFT reconstructed from the masked reflexes in FFT images representing various crystallographic orientations. Assessed interplanar spacings with an accuracy of ±2.5% are also presented in the inset. The area depicted with blue also contains ‘broad’ quasiamorphous regions.

A schematic description of the anodic memristors under study is provided in Figure 1a together with cross-section TEM images. Imaging in the Hf oxide region allows observation of both metallic/oxide interfaces, where no adhesion issues or evidence of mechanical failures of any kind were observed. The HfO_2_/Hf interface appears somewhat smoother when compared to the Pt/HfO_2_ due to the anodic nature of the oxide.

High resolution chemical analysis was performed as well and the obtained mappings are shown in part (b) of Figure 1. The presence of Hf is clear in both oxide and bottom electrode, with a higher density in the metal. The presence of O virtually across the entire analyzed structure is due to contamination and additional oxidation occurring during sample preparation and air-exposure before TEM analysis. However, the highest amount of O is detected in the anodic oxide while Pt is clearly localized in the top electrode. The Pt content in the oxide layer according to EDX measurements is neglectable (Figure 1b). The chemical analysis indicates that all layers are localized as expected from the definition of the MIM structure with the active medium sandwiched between metallic electrodes.

(HR)TEM analysis of the anodic memristor allows observation of HfO_2_ at the atomic level, as presented in Figure 1c. One can see the expected nanocrystalline structure of anodic HfO_2_, and regions with different crystallographic particularities may be observed [20]. A Fourier analysis of the oxide layer regions highlighted in the figure with yellow and blue squares has been performed. The corresponding filtered fast Fourier transforms (FFT) patterns and inverse fast Fourier transforms (IFFT) figures are shown in Figure 1c. Applying the mask to selected reflexes allowed finding the accordant regions in the sample (designated with green numbers and squares in Figure 1c). The interplanar distances were also assessed. With values of 0.31, 0.28, 0.26 nm and in good agreement with literature data, they correspond to (11-1), (111), and (002) planes of the monoclinic HfO_2_ structure, respectively [30]. Additionally, a quasiamorphous region is observed in the (HR)TEM image (marked by a blue square). The boundaries between crystallites (as a well-known diffusion and short-circuit paths) are expected to play a crucial role in the redox process responsible for the memristive switching by facilitating the transport of necessary species under the influence of electric field.

**Figure 2:**
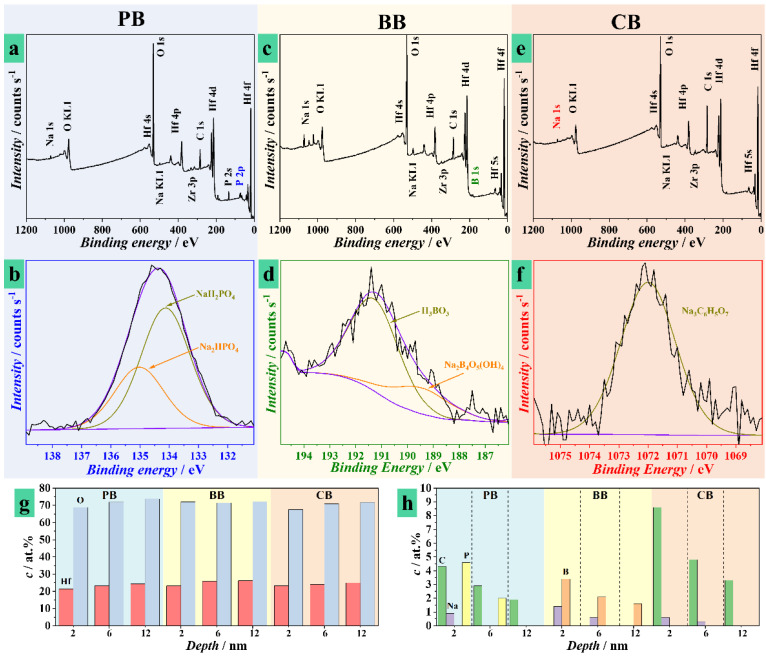
Chemical analysis of HfO_2_ grown in different electrolytes: surface XPS surveys (**a**,**c**,**e**) with corresponding high-resolution analysis of relevant peaks (**b**,**d**,**f**) and quantitative analysis as obtained during depth profiling for Hf and O (**g**) and electrolyte species (**h**).

Chemical analysis of anodic memristors grown in different electrolytes was performed by XPS before depositing the top Pt electrode. In this way, the electrolyte influence on the oxide composition and further electrical behavior could be studied. In Figure 2 these results are presented as surface surveys (a,c,e), high resolution analysis (b,d,f), and quantitative analysis by depth profiling (g,h). The surface surveys show in all cases the presence of Hf and O as constituents of the anodic oxide but also C and Na as contributions from the electrolytes used. Additionally, a small Zr peak may be seen due to the small amount of Zr always present in the Hf sputtering target. The presence of P is evidenced only for the anodic memristor grown in PB, and the 2p peak highlighted in Figure 2a is presented in part (b) of the figure as obtained by high resolution analysis.

Deconvoluted spectra of P 2p reveal signals attributed to phosphates such as NaH_2_PO_4_ and Na_2_HPO_4_ (133–134 eV). The occurrence of B is evidenced in the oxide grown in BB and the 1s peak highlighted in Figure 2c is presented in part (d) of the figure imaged by high resolution analysis. The peak is rather weak but its deconvolution indicates the presence of H_3_BO_3_ and possibly Na_2_B_4_O_5_(OH)_4_ as evidenced by the apparent shoulder peak at lower energies. The memristor grown in CB did not present any additional species as compared to the other ones, since the CB electrolyte uses Na in a similar manner with BB and PB without any additional species. However, in order to investigate the electrolyte incorporation into oxide, high resolution analysis of the Na 1s peak was performed in this case. This is presented in Figure 2f where Na_3_C_6_H_5_O_7_ was identified in the deconvoluted peak.

High resolution analysis of XPS spectra corresponding to C, O, and Hf are presented in the Supplemental Information Figure S1. Deconvoluted spectra of C 1s region generally show three main peaks, which correspond to C–C sp^3^ configuration, C–O–C, O–C=O bonds, and metal carbonates. High resolution O spectra show a peak between 531 and 532 eV representative to a metal carbonate on the surface for all oxides, except those grown in BB. Additionally, there is a second peak around 530 eV specific for metal oxides in all cases. The presence of HfO_2_ is clearly identified in the Hf 4f high resolution spectra independent on the electrolyte used. As is observable in Figure S1, only for oxides grown in PB electrolyte, a chemical shift of both 4f 5/2 and 7/2 peaks is identified. This is attributed to the presence of Hf–O–P bonds due to the fact that PB is the only electrolyte that promotes bonding between electrolyte species and Hf. The data presented in Figure S1 helps emphasizing the electrolyte role into the chemistry of each HfO_2_ memristor.

The presence of electrolyte species was also identified deeper inside the anodic oxide in all cases by high resolution analysis during depth profiling at three different depths. In part (g) of Figure 2 the O and Hf concentrations may be observed. The constant distribution of values across electrolytes and depths suggests a uniform HfO_2_ oxide forming the anodic memristors. The O/Hf content ratio is larger than 2 supporting the conclusion of phosphates, borates (or boric acid), and citrates incorporation into the corresponding anodic Hf oxides. Depth profiles quantifying electrolyte species within oxides anodized in different electrolytes are presented in Figure 2h. In all cases, Na is present close to the surface and its amount decreases in the depth of the memristors. This may be linked to the compositions of anodizing electrolytes, since Na salts were always used. In the PB case, Na incorporation was rather shallow (only down to 2 nm deep) while BB and CB electrolytes allowed Na incorporation deeper down to 6 nm. For obvious reasons, only the BB memristors contained B and its amount is decreasing with the depth, confirming its migration to the surface during the oxide formation process. In a similar manner, P was only detected in the PB memristors, its amount reaching almost 5 at.% close to the surface and vanishing at 12 nm. As previously suggested by the high resolution analysis of the O peak (Figure S1), only the BB memristor did not show any C. The other two memristor types had a decreasing amount of C with increasing depth. If in the case of PB the highest C amount exceeds 4 at.%, this value is doubled in the case of CB. The reason may be found in the electrolyte composition, and such strong difference indicates electrolyte incorporation within the CB memristor. In this case there is no additional species that can be traced (unlike P for PB and B for BB), thus this observation is relevant for concluding upon citrate incorporation.

### 3.2. Electrical Characteristics of Anodic Memristors Grown in Different Electrolytes

**Figure 3:**
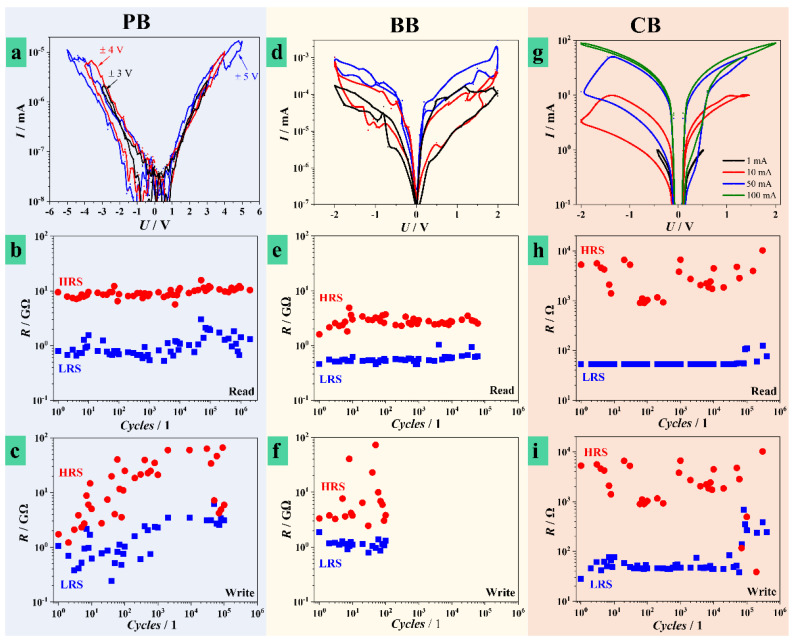
Memristor formation curves together with retention and endurance tests (read/write) for anodic devices grown in PB (**a**–**c**), borate (BB) (**d**–**f**), and citrate (CB) (**g**–**i**) electrolytes.

Device fabrication in different electrolytes was performed in identical conditions (up to 8 V at 100 mVs^−1^) for a direct comparison between memristors. Identification of optimal scan rates and oxide thicknesses was debated in Hf anodization investigations and reported anodic oxides electronic applications, including memristive devices on valve metals [20,31,32,33]. In Figure 3 are presented the main memristive testing results. Memristors were firstly formed by applying voltage in a positive direction under current control (compliance) to prevent irreversible dielectric breakdown and switched, consequently (see experimental section). The switching curves for various electrolytes are presented in Figure 3a,d,g. The memristors were switched during *I-U* sweeps showing bipolar switching behavior. The range of switching voltages was different for different electrolytes. Devices grown in PB (Figure 3a) were switched in a maximum voltage range of ±5.0 V at different current compliances in µA range. However, these limits were not reached and increasing the voltage limit above ±5.0 V resulted in dielectric breakdown. The figure describes three different voltage limits and the highest measured current only reached 10 nA. When switching is limited by the voltage allowing only a very small current to flow, it is likely that only few CFs are formed. However, several tested memristors reached very high current levels (100 mA range) during formation at ±5.0 V suggesting a certain level of instability for PB memristors (see Supplemental Information Figure S2). Additionally, ±5.0 V is a high switching voltage range, considering that the oxide was formed at 8 V.

Due to its band gap of 5.7 eV, the insulating HfO_2_ has previously been assumed as challenging to be formed or switched [20]. The voltage range reported here is lower than those used for Hf memristors with different oxide origins [23,34,35]. The reason for this could be related to the bonding between Hf and P (incorporated from PB electrolyte) which was previously confirmed by XPS measurements in Figure 2. On one hand, this bonding may lead to pining of CFs at constant positions, possibly benefiting the switching features. On the other hand, the bonding may decrease the mobility of O vacancies moving inside the oxide layer [36] and lower the switching and endurance characteristics of PB memristors as compared to devices formed in P free electrolytes (e.g., CB).

Clearer *I*-*U* hysteresis loops were obtained during memristive formation for oxides grown in BB or CB as shown in Figure 3d,g, respectively. Anodization in BB resulted in a maximum switching voltage range of ±2.0 V when the maximum current values reached 1 µA. Higher applied electric fields led to dielectric breakdown. The Hf oxide grown in CB had the most reliable and reproducible memristive formation behavior. The memristors were switched in the same voltage range of ±2.0 V with different current compliances up to 100 mA. High switching reproducibility was observed, indicating that switching characteristics are dependent on electrolyte selection. Multilevel switching tests were performed by stepwise changing of the current compliance after memristive formation. However, this approach rendered reproducible results only for CB, the use of PB or BB led to memristive formation in voltage limitation rather than current, as previously discussed. 

Multilevel switching characteristics of ReRAMs simulate an analog behavior by storing more than one bit per cell (two logic states) when switching between more than two distinct resistance levels [37,38,39]. This is an important concept for memories that is already used in solid-state discs and neuromorphic systems enhancing the overall data density on the storage device but also increasing its complexity [40,41,42]. The existing broad range of electronic applications demand the increase of distinguishable resistance levels, and until now multilevel memristors using Hf oxides are reported as using maximum four levels of resistive states [43]. In this work, at least six distinguishable resistive states were identified in CB, two in BB, and one in PB memristors. This is directly observable in Figure 3a,d,g and also in the Supplemental Information Figure S3, where additional experimental data is provided for the CB case. Formation of the HfO_2_ in CB is highly relevant for the fabrication of anodic memristors with a high number of resistance levels. This may be due to CB being a more abundant source of O vacancies and mobile cations such as Na (see Figure 2e), responsible for the CFs formation that leads to higher memory capacities. Therefore, the larger amount of mobile O vacancies and cations improved the switching of anodic devices formed in CB. 

In Figure 3 are also presented selected curves describing retention and endurance testing for devices anodized in different electrolytes. The variability of the results was assessed by empirically determining confidence bands corresponding to experimental data that are presented in Figure S4. The band edges determination was performed by analyzing the minimum and maximum HRS and LRS values measured from different memristors (5 to 25 different measurements). Stray experimental points (i.e., values measured after or close to memristive failure) were not considered. Retention is given by a reading procedure when the ohmic state of the memristor is cyclically read at low voltages. Endurance is assessed by purposefully switching each device between HRS and LRS a high number of times/cycles during write procedures. Unlike unipolar case, bipolar memristive switching refers to attaining SET and RESET at voltages with alternating polarities [6]. This is the case in the present study. The presence of HRS or LRS is evidenced in the memristive formation curves (Figure 3) by different current values for a given voltage along the hysteresis. Figure 3b,e,h presents resistive states values for PB, BB, and CB memristors, respectively. Since excessive reading eventually lead to device failure, the HRS and LRS values plotted in the graphs come from different devices formed in identical conditions. The failure of the device when performing both endurance and retention measurements is recognized as a sudden increase of the conductance of HRS, leading to a low HRS/LRS ratio. Memristors grown in PB and BB showed rather high resistive states with values in the GΩ range. However, a clear difference of approximately one order of magnitude between HRS and LRS is easily observable. Anodization in BB rendered memristors with a reduced retention as compared to the other electrolytes. They are withstanding only 10^5^ reading cycles whereas PB and CB memristors reached 10^6^ cycles. A clear state difference is observed for the CB case, where LRS values are below 100 Ω and HRS values are in the range of 1–10 kΩ. These values are similar to the ones reported for sputtered Hf oxide memristors, but here the HRS/LRS ratio is slightly higher, exceeding one order of magnitude [44,45]. 

In separate investigations, the endurance of the anodic memristors on Hf was also tested up to 10^6^ cycles. The obtained results from this writing procedure are presented in Figure 3c,f,i. The PB memristors can endure 10^5^ switching cycles but their ohmic state stability is questionable. They likely have a small CF volumetric density, justifying the high resistance values observed in Figure 3b,c. Some of these CF remain irreversible in an oxidized state (HRS) with increasing fatigue, as suggested by the increasing values of both HRS and LRS by at least one order of magnitude before failure. Memristors grown in BB showed very weak endurance, possibly attributed to electrolyte species incorporation that facilitate a premature dielectric breakdown. This idea is consolidated by analyzing the valence band (VB) XPS spectra measured for all anodic memristors under study (presented in Supplemental Figure S5). The VB XPS spectra corresponding to PB and CB memristors have a rather similar shape attributed to the density of states (DoS). However, in an 0–7 eV energy range, the BB memristors show much higher DoS with an abrupt drop at the Fermi level (0 eV). The slope of the DoS near the Fermi level suggests also a rather high DoS in the conduction band. Such behavior is completely insufficient for further electronic implementation of memristors. A clear influence of the electrolyte on the final electrical properties is evidenced. Finally, memristors grown in CB showed better endurance. Their overall behavior suggests a good dynamic during redox processes, as reflected by multilevel switching. Additionally, a high CF density would be expected, given the measured values of HRS and LRS, with good reversibility and reproducibility. The variability of the results may be directly observed in Figure S4. The poorest variability was found for PB memristors, where the confidence bands for HRS and LRS in the writing test strongly overlap, while the CB memristors show clear reproducible separation between confidence bands corresponding to resistive states. 

### 3.3. Temperature Influence on Memristive Switching

Most electronic devices are designed to function in certain temperature conditions and limitations arise for specific applications. As common knowledge examples, a computer motherboard may function properly at temperatures reaching 80 °C, while most RAM modules will show instabilities above 50 °C. Considering the dynamics of the memristive switching, temperature is expected to influence device operation. Many memristors and ReRAM memories were reported to fail at temperatures above 80 °C due to repeated formation and/or deletion of CFs mainly composed of oxygen vacancies. Their assistance in generating an insulating oxide layer of random nature trigger thermal instabilities of CFs [46]. 

**Figure 4:**
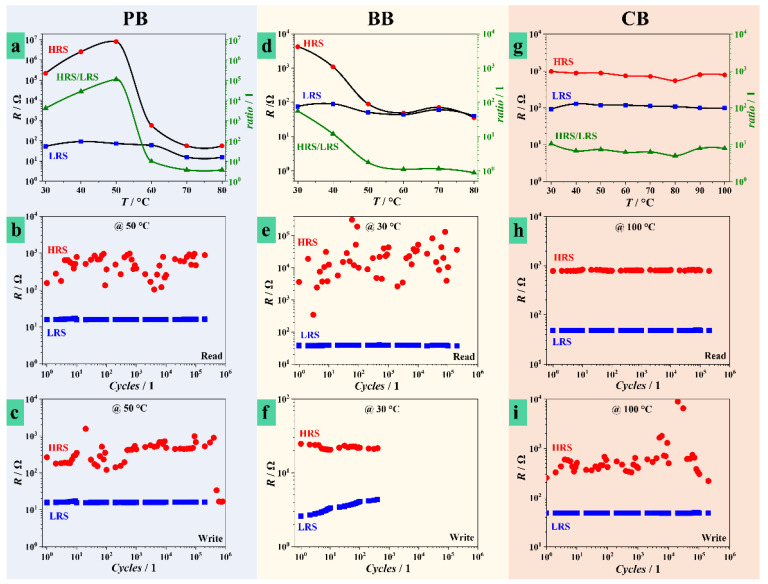
Heat treatment effects on anodic memristive devices, temperature mapped as high resistance states (HRS)/low resistance states (LRS) ratios with retention and endurance (read/write) tests performed at selected optimum temperatures for memristors grown in PB (**a**–**c**), BB (**d**–**f**), and CB (**g**–**i**) electrolytes.

The current study includes a temperature assessment of device performance depending on Hf anodization electrolyte. Figure 4 summarizes the most relevant findings during electrical testing of anodic Hf oxide memristors at temperatures up to 100 °C, and Figure S6 presents the empirically determined confidence bands associated to these measurements. The first row of experimental curves (Figure 4a,d,g) allows comparing values of HRS and LRS measured at different temperatures for different electrolytes. Additionally, the HRS/LRS ratio is temperature-mapped indicating the temperature evolution of each device. The next rows describe the measured resistive states during read and write procedures similar to those described in Figure 3 at selected temperatures above room temperature.

Anodic memristors grown in PB and BB have a strong reaction to applied temperature. Values as low as 30 °C triggered a total change in HRS and LRS values. The previous resistive states measured at room temperature (22 °C, Figure 3) dropped significantly. At 30 °C, HRS of PB memristors are around 100 kΩ and the values increased further with the temperature, reaching a peak at 50 °C. Further, towards 80 °C these values dropped approaching the LRS levels in the 100 Ω range. Since the temperature evolution of LRS was rather uniform, the curve corresponding to HRS/LRS ratio resembles the HRS tendency (Figure 4a). Retention and endurance tests performed on PB memristors formed at the peak temperature of 50 °C (Figure 4b,c) showed an improved stability as compared to room temperature testing (Figure 3). Especially the LRS values are much more reproducible at this temperature. Even though the endurance remained in the same cycle range as before, all states showed a better uniformity and reproducibility up to the failure point. Memristors grown in BB had a similar tendency toward reproducibility starting from 30 °C. The temperature evolution of HRS shows a continuous decrease with increasing temperature, while LRS values remain rather unaffected. In this way, above 50 °C, the memristor effect practically vanishes, since HRS and LRS values become very close. This is better followed with increasing temperature by observing the evolution of HRS/LRS curve. Endurance and retention tests performed at 30 °C confirmed an improvement in the memristive behavior. Read cycles in excess of 10^5^ were possible while the number of write cycles increased by one order of magnitude as compared to the room temperature (Figure 3). Nevertheless, the use of BB for anodic memristive devices remains unacceptable due to unsatisfactory endurance.

The temperature evolution of Hf oxide memristors grown in CB is the most interesting. Up to 100 °C, both HRS and LRS levels remained temperature independent, thus rendering a constant HRS/LRS ratio in the range of one order of magnitude. Retention and endurance tests at various temperatures showed the same results, very similar to those presented in Figure 4h,i corresponding to measurements at 100 °C. This behavior is contrary to the previously described CFs instability. In the case of CB electrolyte, CFs were stable enough to provide invariant values of LRS and HRS within the whole temperature window and their volume density remained constant during the retention check. Interestingly enough, the HRS/LRS ratio also remained constant after cooling down the CB memristor, thus promoting temperature independence. The fabrication of such memristive devices is of extraordinary simplicity, without any need of additional thermal pretreatments. By contrast, memristors formed in PB or BB were highly affected by the increase of temperature. Detailed conceptual studies of memristive devices on valve metals such as Ta and Hf showed a strong influence of the local environment on final properties [34]. After reaching specific temperatures, H_2_O and/or O_2_ can be released from memristors, and further increasing temperature leads to increasing LRS and decreasing HRS towards complete failure of the memristor. In the current study, the memristors grown in CB show totally opposite behavior demonstrating high stability of HRS and LRS by ensuring inherent conductivity from the electrolyte species trapped inside the oxide layer.

**Figure 5:**
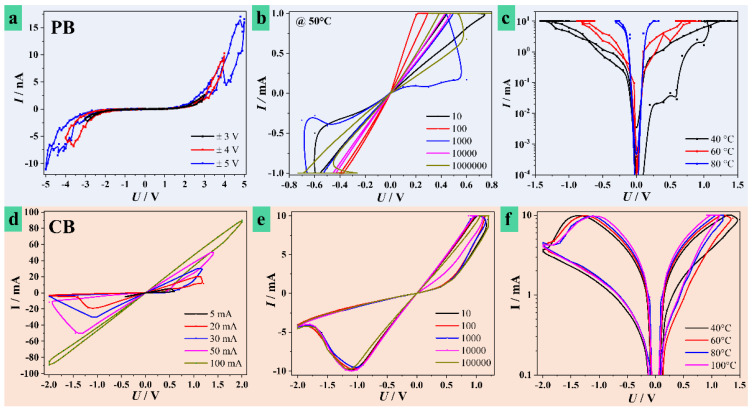
Memristor switching curves with details of endurance testing for increasing cycles numbers and device I-V performance at different temperatures for devices grown in PB (**a**–**c**) and CB (**d**–**f**) electrolytes.

The switching features are regulated by the CF formation, while CFs density and size are dictated by electroformation processes [47,48]. Therefore, formation under the same conditions is crucial for implementation in real systems. In Figure 5, memristive characteristics of devices anodized in the best performing electrolytes and their temperature dependence are compared. Figure 5a summarizes the behavior of PB memristors at room temperature during the switching process, whereas part (b) of the figure describes the cyclization at 50 °C during writing and deleting the CFs. The resistance decrease by the heat treatment is observable in the *I-U* slopes by comparing both figures. The 1000th cycle provides the most conductive value of the LRS and increases the HRS/LRS ratio. Given the erratic behavior of the following cycles, it may be alleged that irreversible filaments are gradually formed after 1000 cycles inducing a certain device instability. This process is typically related to the diffusion of oxygen vacancies into the CF deletion region [46]. A certain material fatigue allows the memristor to reform during its lifetime. Such issues are challenged by using CB, leading to the most stable solid-state electrolyte for memristor fabrication. As previously discussed, the CB memristors exhibit the best reliability and uniformity, independent on temperature, thus no material fatigue inducing reformation is needed. This is probably due to successful reproduction of identical shape and density of CFs through redox processes. Figure 5d,e support this idea for different memristors formed under various current limitations, since repetitive switching during endurance testing had no effect on the *I-U* curves. The temperature independence of CB anodic memristors is further observable in Figure 5f, by contrast to the PB case in Figure 5c, where increasing temperature strongly limits the usable *I-U* ranges, ultimately leading to device failure.

### 3.4. Anodic Memristors Switching Dynamics

**Figure 6:**
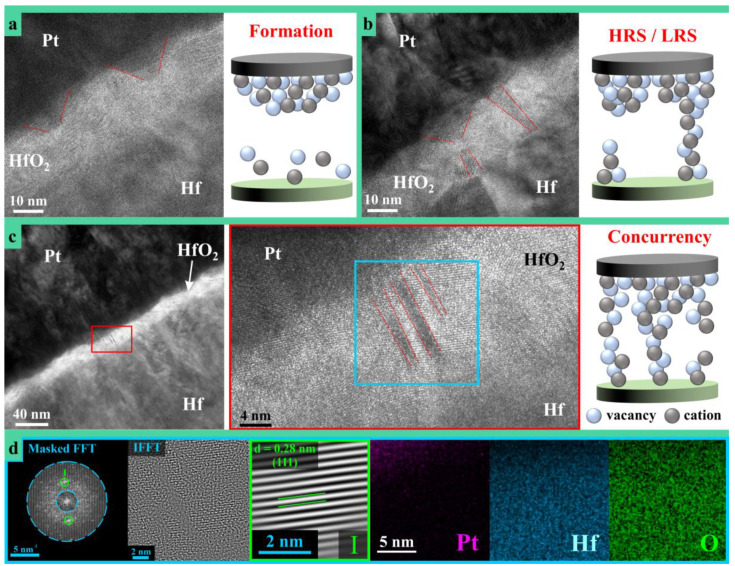
(HR)TEM imaging of an anodic memristor showing (**a**) accumulation zones developed during the formation step, (**b**) complete and interrupted filaments representative for LRS and HRS states, (**c**) concurrent filaments, and (**d**) FFT pattern and filtered IFFT of the region highlighted with a blue square in (**c**). IFFT reconstruction of the masked reflexes in the FFT pattern is presented in a green square. STEM EDX maps (reconstructed using L series of characteristic X-Ray spectrum for Hf and Pt) of the region designated with the blue square are also presented in (**d**).

High resolution transmission electron microscopy was performed for CFs observation in an anodic memristor switched in the HRS before evaluation. The analysis presented in Figure 6 supports the understanding of switching dynamics conditioned by the CFs formation at atomic scale. Additionally, schematic modeling of various stages during the CFs dynamic evolution are also presented in the figure. Trapezoidal shaped accumulation regions can be observed in part (a) of Figure 6 confirming very recent findings [26]. During the memristive formation phase, O vacancies accumulate close to the Pt electrode and their migration disturbs the crystallinity of the HfO_2_. Thus, Figure 6a is a snapshot into the incipient stage of CF formation, with an initial accumulation front in the range of 20 nm, while the filament growth front toward the Hf electrode is in the range of 5 nm as indicated by the dotted lines drawn in the figure. Imaging other regions of the TEM specimen allowed finding a fully developed (i.e., reduced) CF in the vicinity of an incomplete (i.e., partly oxidized) one.

This is observable in Figure 6b and a schematic modeling is also provided. Such situation likely arose due to an incomplete switching to the HRS or due to formation of an irreversible CF. The figure depicts a possible dynamic of filament growth by comparing both entities. The left side filament was likely oxidized during switching to HRS, as suggested by the large trapezoidal shape of the accumulation region aligned with a remainder part of the previous CF. The right-side filament appears complete, oxidation did not fully occur upon switching from LRS. It is possible that the locations of newly formed or deleted CFs are spatially pinned down within the oxide, which explains the ease of memristive switching conditioned by proper electrical formation under current limitation. Moreover, the suggested pinning may be related to additional incorporated electrolyte species and may justify the need for reformation previously discussed during the presentation of Figure 5. Based on this, the schematic modeling of HRS and LRS states presented near the TEM image indicate an electrical contact during LRS and does not exclude the possibility of electron tunneling across the oxidized gap during HRS.

Complete CFs oxidation or reduction is clearly suggested by impedance spectroscopy. Spectra corresponding to memristors grown in different electrolytes are presented as Supplemental Information in Figure S7. Since all impedance spectra are very similar, the dielectric behavior is dictated by HfO_2_, electrolyte species incorporation being insufficient for altering the permittivity. In LRS, all anodic memristors show a frequency independent impedance with an associated phase shift of nearly 0°. This directly proves that all CFs are reduced rendering pure Ohmic behavior. In HRS, all anodic memristors behave as almost ideal capacitors with −1 slopes of impedance spectra and phase shifts approaching −90°. Fitting the experimental data, capacitances of several hundred pF were found with associated parallel resistors in the GΩ range. This demonstrates that CFs are in an oxidized non-conducting state, the memristors acting as pure dielectrics/insulators.

The (HR)TEM images presented in Figure 6c,d show that CF concurrency is encountered in anodic HfO_2_ memristors. Even at lower magnification, several parallel CFs are visible almost connecting the metallic electrodes. Increasing the magnification and resolution of the interest region, three CFs may be observed. Dotted lines are added to the figure for an easier identification. Since no prominent Pt agglomeration localized in CFs region can be observed inside the oxide layer in Figure 6d, it can be assumed that the discussed regions are not affected by Pt diffusion into the anodic oxide. Additionally, the accumulation zones appearing as darker regions connecting the CFs to the Pt electrode also should not be related to Pt diffusion. This is in agreement with recent work on HfO_2_ memristors [26]. From the three CFs visualized, one appears partly interrupted and the one in the middle may suggest a certain degree of amorphization. Likely, most of the current was carried by the middle CF during the LRS. The IFFT image from Figure 6d supports this assumption by hinting an atomic disarray along the middle CF. However, in-depth investigation of this phenomena is still actual. The observed filament concurrency may be responsible for the multilevel switching previously discussed. Depending on the size and atomic constituents of each concurrent filament, their oxidation/reduction may occur for different applied electric fields. The idea of CF spatial pinning is also supported by this image and it may be assumed that the stronger middle filament is also the most fatigued, given its higher atomic disarray. Thus, device failure during endurance testing may occur by transforming such CF into an irreversible dielectric breakdown path.

The CFs dynamics presented in this work follow proposed switching models defined by formation and dissolution of an oxygen deficient region [30], while multiple CFs needs to be formed for reaching the current compliance. During the formation/generation of O vacancies at accumulation regions, metal cations, and electrolyte ions might play a role in defining the CFs size and position due to their limited mobility. Apart from electric field assisted redox reactions or different species inside the oxide that supply the O reservoir controlling the switching dynamics, the environmental O sources from moisture and O_2_ gas need to be considered. This directly relates to different behaviors between anodic memristors thermally treated or formed in different electrolytes. It was previously reported that the switching mechanism may also refer to an electron trapping/detrapping from the Pt interface which can increase or lower the Schottky barrier, thus reducing or increasing the CFs conductance [49]. Additionally, in the present work, the CFs were influenced by electrolyte species incorporation. While Pt diffusion is unlikely due to low operation temperatures, the electron injection from Pt interface may induce the movement of O vacancies accumulated close to it, as well as of additional O vacancies and cations originated from electrolyte (e.g., the CB case). Since the bonding between P and Hf was observed in the case of memristors formed in PB, this may support the statement that CFs were pinned at constant positions exactly due to the formation of Hf-P bonds dictating certain fixed size and shape of CFs. This will prevent the sudden diffusion of O vacancies and consequently CFs rupture. It may be concluded that the synergistic effect of these mechanisms affected the conductance of CFs and accordingly improved the electrical properties of CB and PB devices. However, future studies on optimization of size and shape of CFs are crucial in order to achieve defect engineered memristors.

## 4. Conclusions

In the present work anodic HfO_2_ memristors grown in different electrolytes are characterized at fundamental and device levels. Incorporation of PB, BB, and CB species into the anodic memristors was confirmed by XPS, while the conservation of crystalline structure of HfO_2_ was demonstrated by HR-TEM. Upon electroforming, memristors were switched and their characteristics were compared as a function of electrolyte. Retention and endurance tests were performed on memristors by using read and write cycles up to 10^6^. The use of BB resulted in the weakest memristive performance. Memristors grown in PB withstand 10^6^ reading cycles and 10^5^ writing cycles before device failure, but showed very high ohmic state values in the range of GΩ. The use of CB electrolyte clearly demonstrated superior memristors. Multilevel switching was also achieved together with high read/write cycling. Low thermal treatments applied to the memristors showed a direct influence on the resistive levels and switching behavior. Memristors grown in BB and PB had their HRS values around 1 kΩ after heating up to 50 °C. The use of CB rendered memristors with electrical characteristics independent of temperatures up to 100 °C. All parameters investigated remained constant before, during, and after applying the thermal treatment, indicating robustness and reliability for use in real-life applications. The switching dynamic of HfO_2_ memristors was discussed at fundamental levels by analyzing HR-TEM images of memristors in HRS. Apart from the switching models suggested by the atomic scale study visualizing oxidized and reduced filaments, a concurrency of CFs was additionally suggested. This was used to ground the multilevel switching mechanism in HfO_2_ and to elucidate mechanisms related to device failure at the end of their lifetime.

## Data Availability

The data presented in this study are available on request from the corresponding author.

## Supplementary Materials

The following are available online at https://www.mdpi.com/2079-4991/11/3/666/s1, Figure S1: High resolution XPS spectra for C, O, and Hf as measured on the surface of HfO_2_ memristors grown in different electrolytes, Figure S2: Atypical formation curves with very high current compliances for Hf oxide memristors grown in PB, Figure S3: Multilevel switching of Hf anodic memristors grown in CB: (a) *I-U* and (b) *R-U* characteristics for various current compliances, Figure S4: Empirically determined confidence bands for the variability of HRS and LRS values depending on electrolyte, Figure S5: Superimposed high resolution XPS valence band spectra for various anodic memristors with highlighted region immediately above Fermi level, Figure S6: Empirically determined confidence bands for the variability of HRS and LRS values at different temperatures depending on electrolyte, Figure S7: Bode representations of impedance spectroscopy data performed on anodic memristors grown in various electrolytes. The impedance (a) and phase shift (b) are presented for different switching states.
