# Supplementary material for: Electrolyte-Dependent Modification of Resistive Switching in Anodic Hafnia
Source: Nanomaterials (Basel). 2021 Mar 8;11(3):666. doi: 10.3390/nano11030666 (PMC8001223; doi:10.3390/nano11030666)
Supplement: Supplementary file 1 [file nanomaterials-11-00666-s001.pdf]

nanomaterials

Supplementary Materials: Electrolyte-Dependent Modification of Resistive Switching in Anodic Hafnia

Ivana Zrinski [1], Cezarina Cela Mardare [1], Luiza-Izabela Jinga [2], Jan Philipp Kollender [1,3], Gabriel Socol [2], Alexey Minenkov [4], Achim Walter Hassel [1] and Andrei Ionut Mardare [1,*]

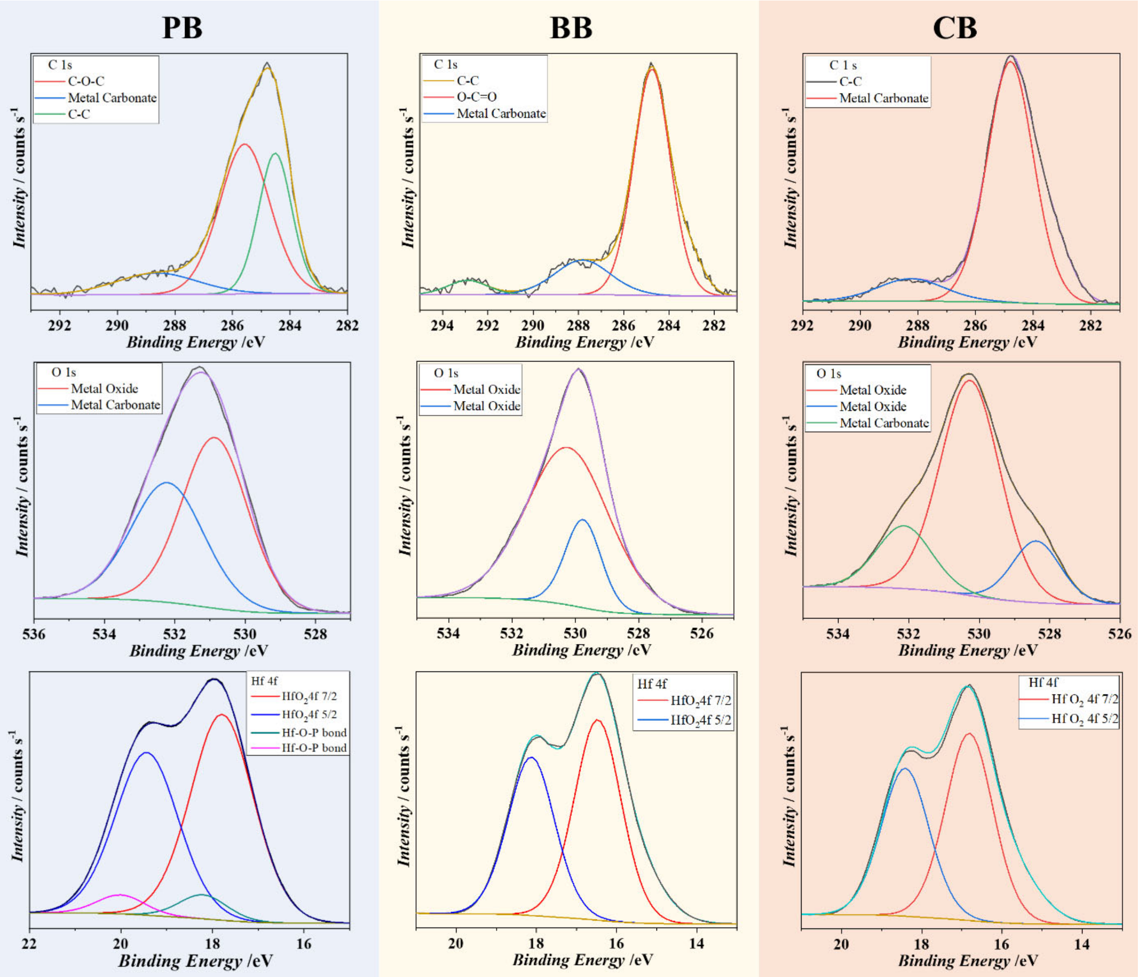

Figure S1. High resolution XPS spectra for C, O and Hf as measured on the surface of HfO_2 memristors grown in different electrolytes.

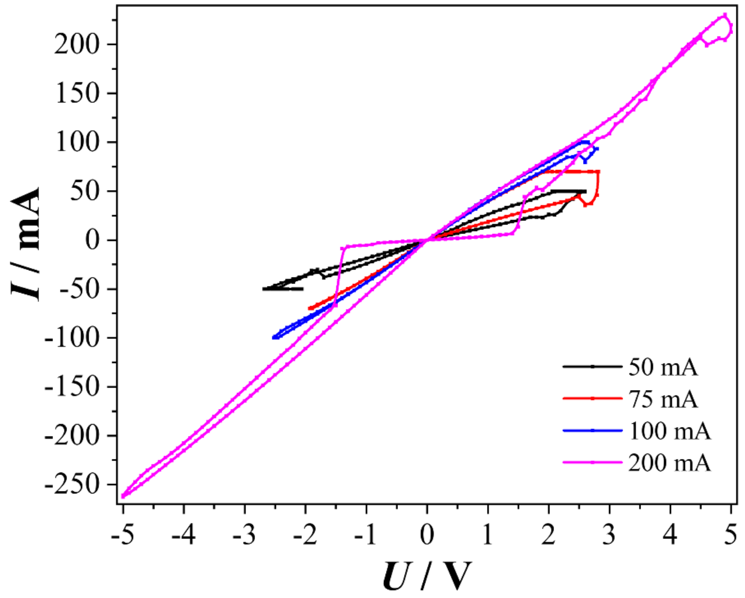

Figure S2. Atypical formation curves with very high current compliances for Hf oxide memristors grown in PB.

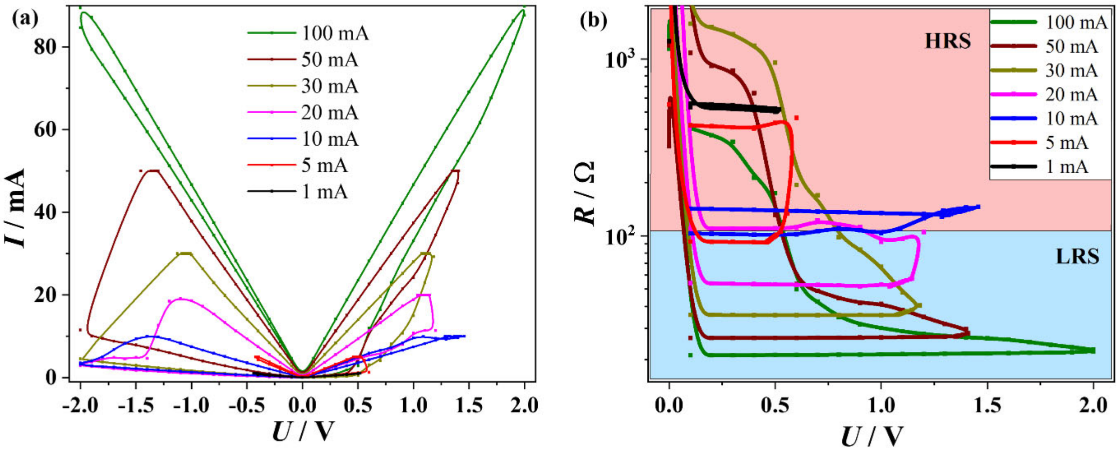

Figure S3. Multilevel switching of Hf anodic memristors grown in CB: (**a**) *I-U* and (**b**) *R-U* characteristics for various current compliances.

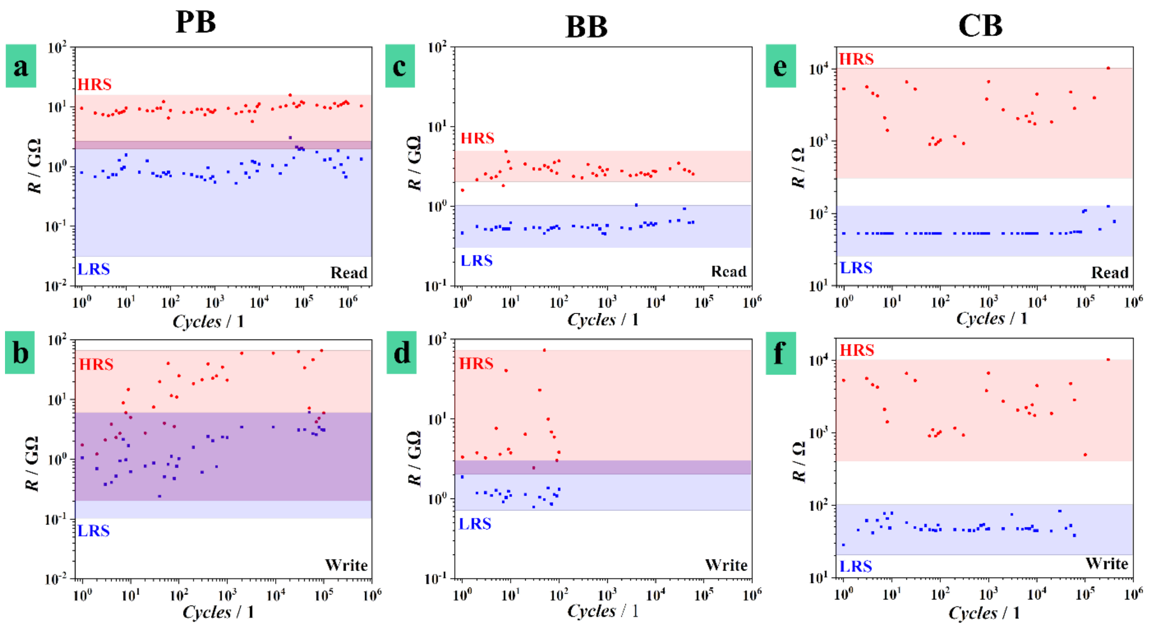

Figure S4. Empirically determined confidence bands for the variability of HRS and LRS values depending on electrolyte.

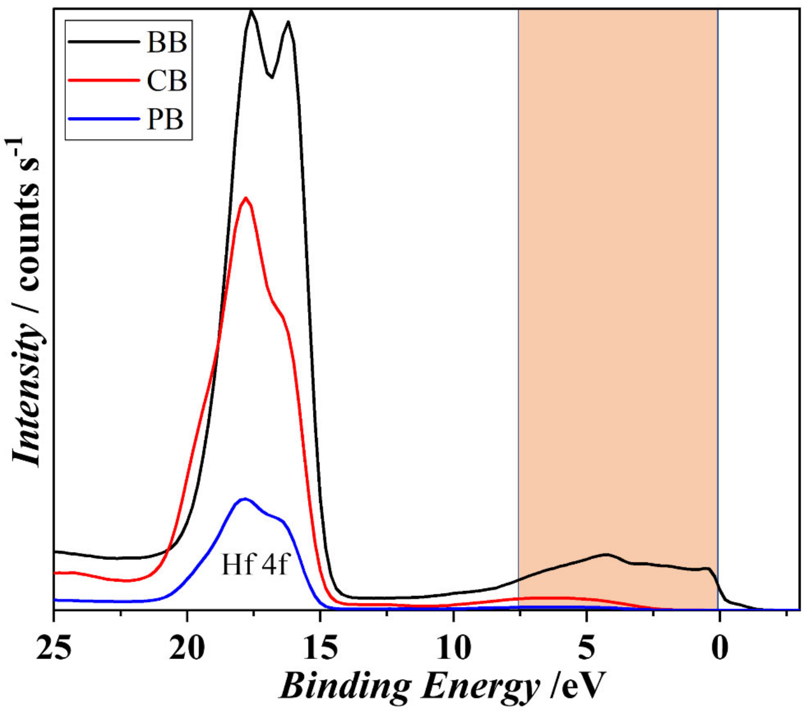

Figure S5. Superimposed high resolution XPS valence band spectra for various anodic memristors with highlighted region immediately above Fermi level.

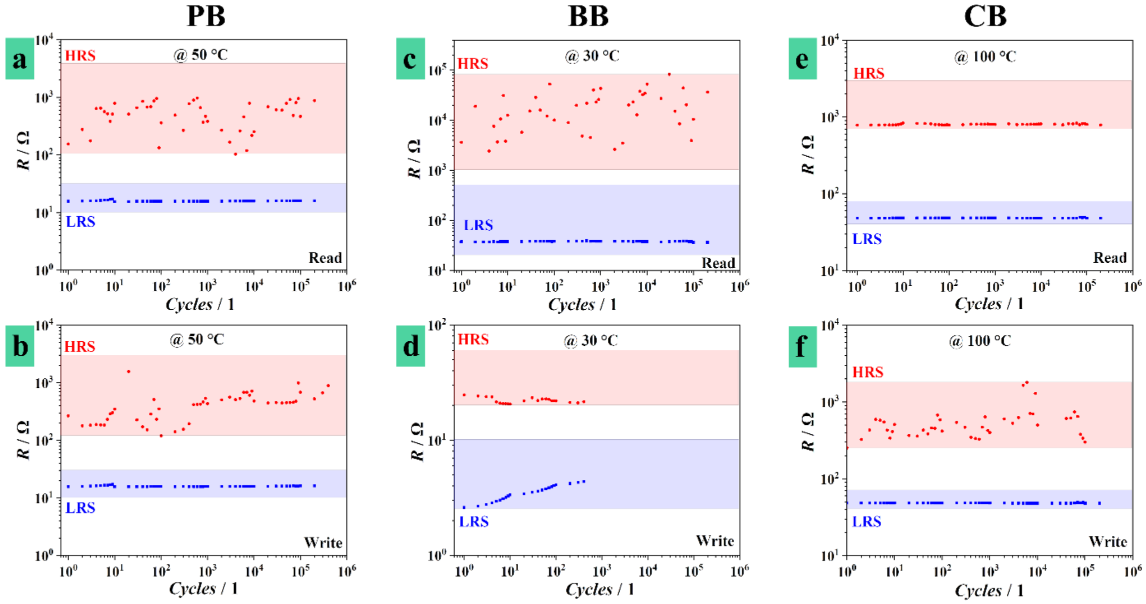

Figure S6. Empirically determined confidence bands for the variability of HRS and LRS values at different temperatures depending on electrolyte.

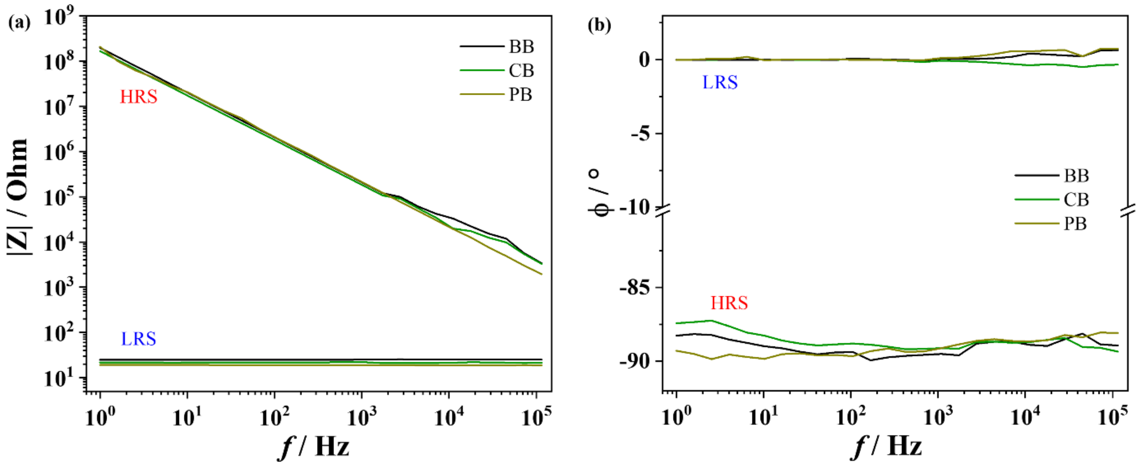

Figure S7. Bode representations of impedance spectroscopy data performed on anodic memristors grown in various electrolytes. The impedance (**a**) and phase shift (**b**) are presented for different switching states.
